# Supplementary material for: Longitudinal localization of leukaemic stem cells between the metaphysis and central marrow governs their behaviour
Source: Nat Cell Biol. 2026 Apr 24;28(5):890–902. doi: 10.1038/s41556-026-01939-3 (PMC13179134; doi:10.1038/s41556-026-01939-3)
Supplement: Supplementary file 1 — Reporting Summary [file 41556_2026_1939_MOESM1_ESM.pdf]

Corresponding author(s): Xunlei Kang

Last updated by author(s): Feb 25, 2026

Reporting Summary

Nature Portfolio wishes to improve the reproducibility of the work that we publish. This form provides structure for consistency and transparency in reporting. For further information on Nature Portfolio policies, see our Editorial Policies and the Editorial Policy Checklist.

Statistics

For all statistical analyses, confirm that the following items are present in the figure legend, table legend, main text, or Methods section.

n/a	Confirmed	
☐	☒	The exact sample size (*n*) for each experimental group/condition, given as a discrete number and unit of measurement
☐	☒	A statement on whether measurements were taken from distinct samples or whether the same sample was measured repeatedly
☐	☒	The statistical test(s) used AND whether they are one- or two-sided<br>*Only common tests should be described solely by name; describe more complex techniques in the Methods section.*
☒	☐	A description of all covariates tested
☐	☒	A description of any assumptions or corrections, such as tests of normality and adjustment for multiple comparisons
☐	☒	A full description of the statistical parameters including central tendency (e.g. means) or other basic estimates (e.g. regression coefficient) AND variation (e.g. standard deviation) or associated estimates of uncertainty (e.g. confidence intervals)
☐	☒	For null hypothesis testing, the test statistic (e.g. *F*, *t*, *r*) with confidence intervals, effect sizes, degrees of freedom and *P* value noted<br>*Give P values as exact values whenever suitable.*
☒	☐	For Bayesian analysis, information on the choice of priors and Markov chain Monte Carlo settings
☒	☐	For hierarchical and complex designs, identification of the appropriate level for tests and full reporting of outcomes
☒	☐	Estimates of effect sizes (e.g. Cohen's *d*, Pearson's *r*), indicating how they were calculated

Our web collection on statistics for biologists contains articles on many of the points above.

Software and code

Policy information about availability of computer code

Data collection	Data collection was performed using commercial software only. No custom code was used.<br>• Flow cytometry raw data were acquired on an LSRFortessa X-20 cell analyzer (BD Biosciences) using FACSDiva v8.0 acquisition software.<br>• Immunofluorescence images were captured on a Keyence BZ-X800 fluorescence microscope using BZ-X Viewer v1.3.1.<br>• Single-cell library generation and sequencing were performed on a 10x Genomics Chromium Controller (Single Cell 3′ v2 kit) and an Illumina NovaSeq 5000 (75-cycle kit). Raw base calls were converted to FASTQ files using bcl2fastq v2.20.<br>• Total RNA from FACS-sorted BM AML cells was used to prepare TruSeq stranded poly(A)+ libraries, which were sequenced on an Illumina NextSeq 500.
Data analysis	Data analysis was performed using commercially available and open-source software only. No custom code was used.<br>• RNA-Seq data:Reads were quality-checked, trimmed, aligned to mm10 with STAR, counted by featureCounts, and differential expression was called using DESeq2.<br>• scRNA-seq preprocessing (demultiplexing, alignment, UMI counting) with Cell Ranger Single Cell Software Suite v3.0.2 (10x Genomics).<br>• Downstream single-cell analysis in R v4.0.5 using the Seurat v3.2 package (normalization, clustering, TSNE).<br>• Statistical analyses (t-tests, ANOVA, nonparametric tests) and plotting in GraphPad Prism v9.0.<br>• Cytokine bead assay data were analyzed with LEGENDplex Data Analysis software v8.0 (BioLegend).<br>• Imaging quantification was carried out with Keyence BZ-X800 analyzer software v1.2.<br>• Flow cytometry data were analyzed using FlowJo v10 (BD Biosciences).

For manuscripts utilizing custom algorithms or software that are central to the research but not yet described in published literature, software must be made available to editors and reviewers. We strongly encourage code deposition in a community repository (e.g. GitHub). See the Nature Portfolio guidelines for submitting code & software for further information.

Data

Policy information about availability of data

All manuscripts must include a data availability statement. This statement should provide the following information, where applicable:

- Accession codes, unique identifiers, or web links for publicly available datasets
- A description of any restrictions on data availability
- For clinical datasets or third party data, please ensure that the statement adheres to our policy

Sequencing data generated in this study are publicly available in the NCBI Sequence Read Archive (SRA) under BioProject accession PRJNA1077712. RNA-seq data from the Dpp4^{+}/$^{+}$ and Dpp4^{-}/$^{-}$ AML model used in this study are available under SRA accession SRP323430. Source data are provided with this study. All other data supporting the findings of this study are available within the paper and its Supplementary Information files.

Research involving human participants, their data, or biological material

Policy information about studies with human participants or human data. See also policy information about sex, gender (identity/presentation), and sexual orientation and race, ethnicity and racism.

Reporting on sex and gender	N/A
Reporting on race, ethnicity, or other socially relevant groupings	N/A
Population characteristics	N/A
Recruitment	N/A
Ethics oversight	All animal experiments were approved by the Institutional Animal Care and Use Committee (IACUC) of the University of Missouri (protocol 65384).

Note that full information on the approval of the study protocol must also be provided in the manuscript.

Field-specific reporting

Please select the one below that is the best fit for your research. If you are not sure, read the appropriate sections before making your selection.

☒ Life sciences ☐ Behavioural & social sciences ☐ Ecological, evolutionary & environmental sciences

For a reference copy of the document with all sections, see nature.com/documents/nr-reporting-summary-flat.pdf

Life sciences study design

All studies must disclose on these points even when the disclosure is negative.

Sample size	No statistical methods were used to predetermine sample sizes. Sample sizes were selected based on prior experience with the AML transplantation and bone marrow niche models and are comparable to those reported in previous publications using similar experimental systems (Wang et al., Cell Reports, 2023; PMID: 36807138). The chosen sample sizes are consistent with established standards in the field and were sufficient to detect biologically meaningful differences with appropriate statistical tests.
Data exclusions	No data or animals were excluded from any analyses. All collected samples and experimental measurements are included in the reported results.
Replication	All key findings were reproduced in at least three independent experiments using distinct biological replicates (separate cohorts of mice or independent cell preparations). Consistent results were obtained across these replicates.
Randomization	After genotyping, mice were randomly assigned to experimental or control groups. Randomization was performed by a laboratory member not involved in data acquisition, using a random-number generator.
Blinding	Investigators performing data acquisition and image quantification were not blinded to genotype when handling animals (genotype-specific treatments required identifiable handling), but all data analyses (flow-cytometry gating, image quantification and statistical testing) were conducted with the analyst blinded to group allocation.

Reporting for specific materials, systems and methods

We require information from authors about some types of materials, experimental systems and methods used in many studies. Here, indicate whether each material, system or method listed is relevant to your study. If you are not sure if a list item applies to your research, read the appropriate section before selecting a response.

Materials & experimental systems

n/a	Involved in the study
☐	☒ Antibodies
☒	☐ Eukaryotic cell lines
☒	☐ Palaeontology and archaeology
☐	☒ Animals and other organisms
☒	☐ Clinical data
☒	☐ Dual use research of concern
☒	☐ Plants

Methods

n/a	Involved in the study
☒	☐ ChIP-seq
☐	☒ Flow cytometry
☒	☐ MRI-based neuroimaging

Antibodies

Antibodies used

Anti-CD3e-PE/Cyanine5 (clone 17A2, BioLegend, #100310, 1:200, flow cytometry), Anti-Ly6G/Ly6C (Gr-1)-PE/Cyanine5 (clone RB6-8C5, BioLegend, #108410, 1:200, flow cytometry), Anti-CD11b-PE/Cyanine5 (clone M1/70, BioLegend, #101210, 1:200, flow cytometry), Anti-CD45R-PE/Cyanine5 (clone RA3-6B2, BioLegend, #103210, 1:200, flow cytometry), Anti-Ter-119-PE/Cyanine5 (clone TER-119, BioLegend, #116210, 1:200, flow cytometry), Anti-CD117 (c-Kit)-APC (clone 2B8, BioLegend, #105812, 1:200, flow cytometry), Anti-Sca-1-PE-Cy7 (clone D7, BioLegend, #108114, 1:200, flow cytometry), Anti-CD150-PE (clone TC15-12F12.2, BioLegend, #115904, 1:200, flow cytometry), Anti-CD48-APC/Cyanine7 (clone HM48-1, BioLegend, #103432, 1:200, flow cytometry), Anti-Ki67-FITC (clone 16A8, BioLegend, #652410, 1:200, flow cytometry), Anti-CD16/32-PE (clone 93, BioLegend, #101308, 1:200, flow cytometry), Anti-CD34-FITC (clone RAM34, eBioscience, #11-0341-82, 1:200, flow cytometry), Anti-CD127-APC/Cyanine7 (clone A7R34, BioLegend, #135040, 1:200, flow cytometry), Anti-CD135-Brilliant Violet 421 (clone A2F10, BioLegend, #135314, 1:200, flow cytometry), Annexin V (BioLegend, #640941, 1:20, apoptosis assay), propidium iodide (BioLegend, #421301, 1:50, apoptosis assay), and Hoechst 34580 (BD Pharmingen, #565877, nuclear staining). For immunofluorescence, anti-Endomucin (goat polyclonal, R&D Systems, #AF4666, 1:100), Biotin anti-mouse Lineage Panel (clone 145-2C11; RB6-8C5; RA3-6B2; Ter-119; M1/70 BioLegend # 133307, 1:200), Biotin anti-mouse IL-7Rα (clone A7R34, BioLegend,#135006, 1:200), Biotin anti-mouse Sca-1 (clone D7, BioLegend,#108104, 1:200), PE-conjugated anti-CD34 (clone RAM34, BioLegend, 1:200), Alexa Fluor 647-conjugated anti-c-Kit (clone 2B8, Invitrogen, 1:200), and anti-GPC3 (rabbit polyclonal, Abcam, #ab216606, 1:200) were used. Secondary antibodies included donkey anti-goat Alexa Fluor® 555 (Invitrogen, 1:500), goat anti-rabbit Alexa Fluor® 750 (Invitrogen, 1:500), and Brilliant Violet 421®-conjugated streptavidin (BioLegend, 1:500). For western blotting, Phospho-p44/42 MAPK (Erk1/2) (Thr202/Tyr204) (clone 20G11, Cell Signaling Technology, #75796S, 1:1000), Phospho-NF-κB p65 (clone 93H1, Cell Signaling Technology, #3039S, 1:1000), Phospho-STAT3 (Tyr705) (clone D3A7, Cell Signaling Technology, #9145S, 1:2000), Phospho-p38 MAPK (clone D3F9, Cell Signaling Technology, #4092S, 1:1000), and β-actin (clone C4, Santa Cruz Biotechnology, #sc-47778, 1:2000) were used. HRP-conjugated anti-rabbit and anti-mouse secondary antibodies (R&D Systems, #HAF007 and #HAF005, respectively, 1:1000) were used for detection with a chemiluminescent substrate (Invitrogen).

Validation

All primary antibodies used in this study were commercially obtained and validated by the manufacturers for the indicated applications (flow cytometry, immunofluorescence, or western blot). Validation data, including specificity testing and performance validation, are available on the manufacturers' websites. In addition, the specificity of key antibodies was supported by expected molecular weight detection (for western blot) or appropriate staining patterns consistent with known biological expression profiles.

Animals and other research organisms

Policy information about studies involving animals; ARRIVE guidelines recommended for reporting animal research, and Sex and Gender in Research

Laboratory animals

All animal experiments were performed in accordance with institutional guidelines and were approved by the Institutional Animal Care and Use Committee (IACUC) of the University of Missouri (protocol number 65384). C57BL/6NCrl mice (Mus musculus) were obtained from Charles River Laboratories, Inc. Dpp4 flox/flox mice were generated by breeding targeted C57Bl/6NTac-DPP4tm1a Wtsi/Ics mice (European Mouse Mutant Cell Repository, EUCOMM) with 129S4/Bl6-Gt (ROSA) 26Sortm2(FLP*) Sor/J (stock #012930, The Jackson Laboratory, Bar Harbor, ME). The offspring were further crossed with Vav-iCre mice (stock #018968, The Jackson Laboratory, Bar Harbor, ME), to generate Dpp4 fl/fl ; Vav-Cre mice. N-cad-tdTomato (N-cad-TdT), N-cad-CreER and Gpc3 fl/fl strains were generated by Dr. Linheng Li's lab. Cxcl12 fl/fl (stock #022457), Scf fl/fl (stock #017861) and Nestin-CreER (stock #016261) mice were purchased from Jackson Lab. To induce expression of Cre-ER recombinase, mice received tamoxifen via intraperitoneal injection (Sigma, 75 mg tamoxifen/kg body weight) as described.

Wild animals

The study did not involve wild-caught or non–laboratory animals.

Reporting on sex

Both sexes were included. No sex-specific differences were observed, and sex was not treated as a variable in statistical analyses.

Field-collected samples

The study did not use any field-collected tissues or organisms.

Ethics oversight

All mouse strains used in this study had a C57BL/6 genetic background. Both male and female mice aged 6–10 weeks were used unless otherwise specified. Animals were randomly assigned to experimental groups based on genotyping results. Investigators were blinded to group allocation during data analysis but not during experimental procedures. Sample sizes for each experiment are

detailed in figure legends. Mice were housed in a specific pathogen–free facility under a 12-hour light/12-hour dark cycle at an ambient temperature of 20–24 °C and relative humidity of 40–60%, with ad libitum access to food and water.

Note that full information on the approval of the study protocol must also be provided in the manuscript.

Plants

Seed stocks	N/A
Novel plant genotypes	N/A
Authentication	N/A

Flow Cytometry

Plots

Confirm that:

☒ The axis labels state the marker and fluorochrome used (e.g. CD4-FITC).

☒ The axis scales are clearly visible. Include numbers along axes only for bottom left plot of group (a 'group' is an analysis of identical markers).

☒ All plots are contour plots with outliers or pseudocolor plots.

☒ A numerical value for number of cells or percentage (with statistics) is provided.

Methodology

Sample preparation	Peripheral blood (EDTA), bone marrow, spleen and liver were harvested and filtered through a 70 µm strainer. Red cells were lysed with ACK buffer, and remaining cells washed and resuspended in PBS + 2% FBS + 2 mM EDTA.
Instrument	Data were acquired on a BD LSRFortessa X-20 cell analyzer (BD Biosciences).
Software	Acquisition: FACSDiva v8.0<br>Analysis: FlowJo v10, using consistent gating templates across all replicates.
Cell population abundance	Each panel reports the percentage of marker-positive cells (mean ± SEM) from ≥ 5 biologically independent mice per group (with technical duplicates).
Gating strategy	1. FSC/SSC to exclude debris.<br>2. Singlet gate (FSC-A vs FSC-H).<br>3. Live/dead exclusion (PI- or Annexin V-negative).<br>4. Lineage (CD3ε, Gr-1, CD11b, B220, Ter-119) to identify Lin⁻ cells.<br>5. L-GMP:GFP+IL-7R–Lin–Sca-1–c-Kit+CD34+FcRII/III+<br>6. Bone marrow niche cells: 7AAD-GFP-CD45-

☒ Tick this box to confirm that a figure exemplifying the gating strategy is provided in the Supplementary Information.
