# Supplementary material for: Longitudinal localization of leukaemic stem cells between the metaphysis and central marrow governs their behaviour
Source: Nat Cell Biol. 2026 Apr 24;28(5):890–902. doi: 10.1038/s41556-026-01939-3 (PMC13179134; doi:10.1038/s41556-026-01939-3)

Fig. 5k and Extended Data Fig. 8a – p-ERK

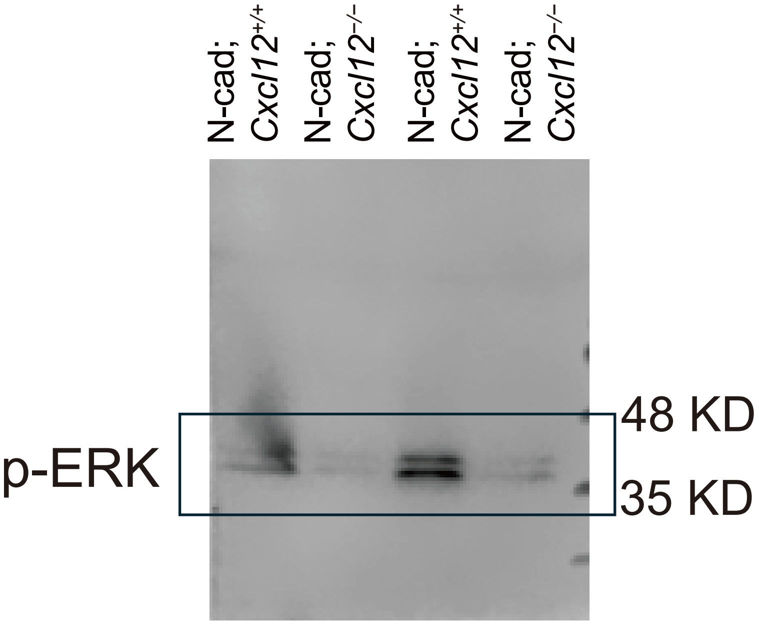

Fig. 5k and Extended Data Fig. 8b – p-NFκB

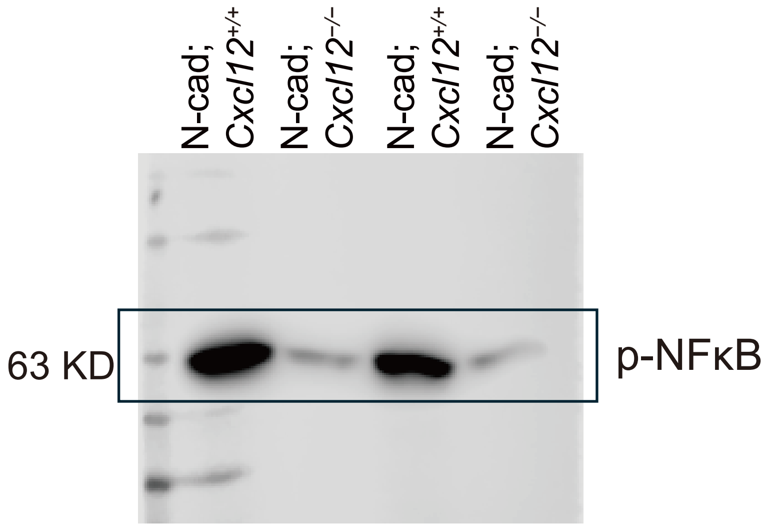

Fig. 5k and Extended Data Fig. 8c – p-Stat3

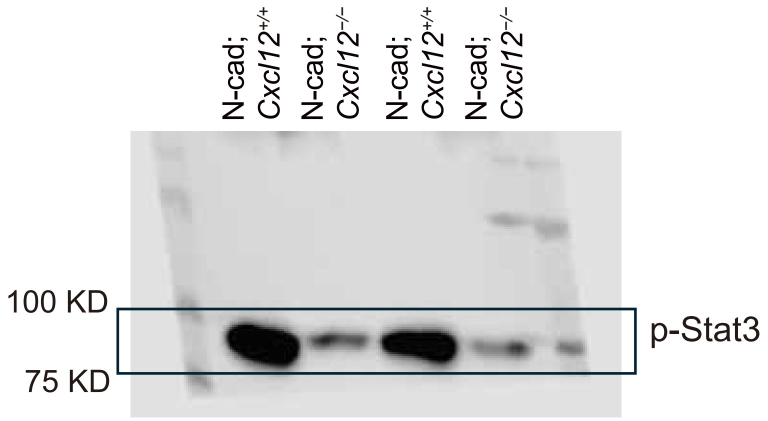

Fig. 5k and Extended Data Fig. 8d – p-p38 MAPK

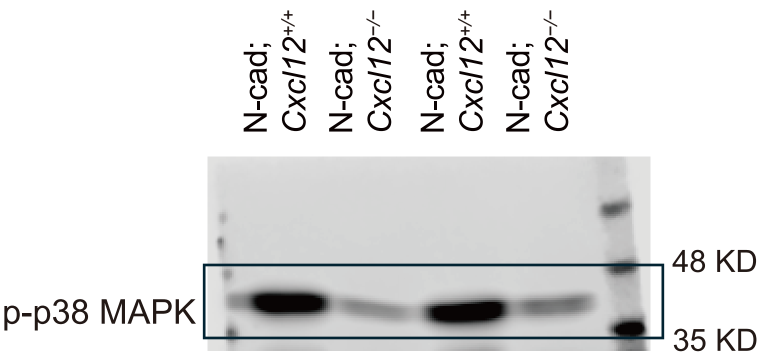

Fig. 5k and Extended Data Fig. 8e – β-Actin

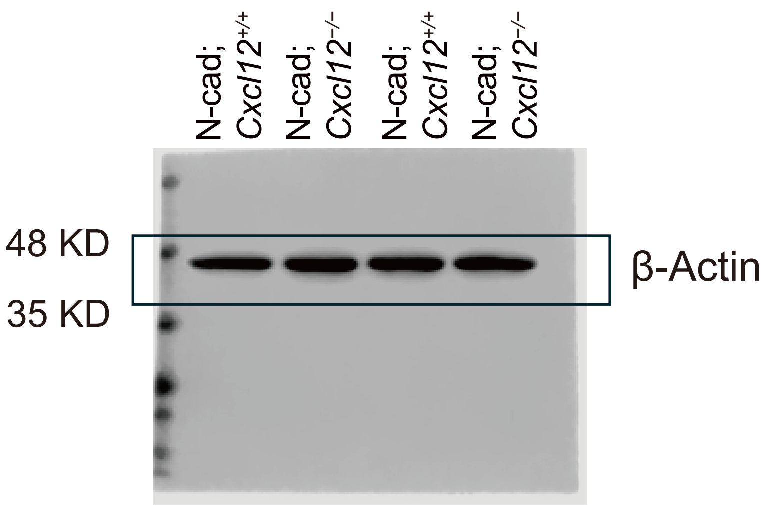

Supplement: Supplementary file 15 — Unprocessed western blots for Fig. 5k and Extended Data Fig. 8. [file 41556_2026_1939_MOESM15_ESM.pdf]
